# Perception of Medical University Members From Nutritional Health in the Quran

**DOI:** 10.5812/ircmj.10846

**Published:** 2014-04-05

**Authors:** Shahin Salarvand, Yadollah Pournia

**Affiliations:** 1 Faculty of Nursing and Midwifery, Hepatitis Research Center, Lorestan University of Medical Sciences, Khorramabad, IR Iran; 2 Faculty of Medicine, Lorestan University of Medical Sciences, Khorramabad, IR Iran

**Keywords:** Nutritional Sciences, Quran, Faculty Member, Phenomenology

## Abstract

**Background:**

Desirable health is impossible without good nutrition, and Allah has addressed us on eating foods in 118 verses.

**Objectives:**

This study aimed to describe the medical university faculty members’ perceptions of nutritional health in the Quran, revealing the important role of faculty members.

**Materials and Methods:**

This qualitative study was conducted with a phenomenological approach. Homogeneous sampling was performed in a final sample size of 16 subjects. The Colaizzi's phenomenological method was applied for data analysis.

**Results:**

Three main categories were extracted from the data analysis, including the importance of nutrition in the Quran (referring to certain fruits, vegetables and foods, illustrating and venerating the heavenly ones, nutritional recommendations, revealing the healing power of honey and the effects of fruits and vegetables on physical and social health); reasons of different foods being lawful (halal) and unlawful (haram) (religious slaughter, wine, meats, consequences of consuming haram materials, general expression of halal and haram terms); and fasting (fasting and physical health, fasting and mental health).

**Conclusions:**

What has been mentioned in the Quran is what scientists have achieved over the time, since the Quran is governed by logic. Although we do not know the reasons for many things in the Quran, we consider it as the foundation.

## 1. Background

Desirable health is impossible without good nutrition (1). Selection of foods naturally reflects the aspects of lifestyle, culture, religion, diet, and health (2). Nutrition is not only a means of preventing diseases, but plays an important role in improving the health of individuals and communities (1). Diets have experienced considerable qualitative and quantitative changes with different rates all over the world (3). Therefore, proper nutrition interventions should be applied to improve the human health (4). On the other hand, in Islam, the Quran and the Sunnah are the main sources of the rules and principles that guide the lives of Muslims and offer policies and recommendations as responses to the concurrent health and social problems (5). Muslims consider the divine rules in every aspect of life. For the followers of Islam, there is a complete code of nutritional rules in the Holy Quran (6). The recommendations on nutrition presented by Islam not only lead to physical health, but guarantee the mental health. One of the most important determinants of health is following the health teachings of Islam on eating and drinking (7). Moreover, a lot of attention has been paid to food, so that the Quran says: “Then let man look to his food, (Abasa:24)” (1). The “all-provider” term as one of the attributes of Allah, Allah’s swearing on foods (swearing on figs and olives), and requests of manna and foods by Isa (Jesus) and Musa (Moses) each confirm the importance of food and nutrition (8). There are many verses in the Quran about foods. In the 118 verses, Allah has spoken to us about foods and eating. This indicates the importance of nutrition from the perspective of Islam since everything we eat or drink influences not only the body but also the soul (9). In this holy book, an emphasis has been placed on some nutrients, and their benefits are occasionally described (Muhammad:15, Al-Baqarah:57, Al-An’am:99, An-Nahl:67) (1).

Awareness of the nutritional rules is essential for dietitians in different cultures (10). Although some aspects of Islam have been studied in researches in a limited way, and the surprising role of this divine religion in promoting mental health, health-promoting behaviors, longevity, etc. has been known, Muslims’ knowledge and practice of these nutritional rules have not been investigated sufficiently (11). On the other hand, bad nutritional habits and their consequences have increased in societies, particularly among young people. In addition, in the present world, the share of scientific production by the Muslim elites in the international arena is very small, and in the Islamic sciences it is even smaller (12).

## 2. Objectives

Since the faculty members in medical science universities deal closely with health and nutrition, this study was conducted to investigate their perceptions of nutritional health in the Quran.

## 3. Materials and Methods

A descriptive phenomenological approach was applied in this qualitative study. A qualitative approach was selected to obtain rich information about the faculty members’ perceptions from the nutritional health in the Quran. The phenomenological approach deals with revealing the nature of meanings hidden in experiences (13). Therefore, considering participants’ viewpoints is one of the characteristics of a qualitative study (14). Unstructured in-depth interviews were performed to collect data from the faculty members in a 9-month period. The samples were selected using homogeneous sampling. The faculty members who had experienced the recitation of the Quran and meditation on its verses, and were willing to participate in the interviews were included. The faculty members who did not have these criteria were excluded. Characteristics of the samples are presented in Table 1.

The information-rich samples were selected and provided with necessary information. After the participants completed the informed consents, time and place of the interviews were arranged. The interviews were conducted in the faculty members’ rooms without the presence of other persons, or in a quiet place. First, the main question was asked: “What is your perception of the nutritional health in the Quran?” Then, more questions were prepared for the following sessions based on the condition and the participant’s answers. All the interviews were performed by one person (the main researcher) recorded on a voice recorder. The interviewer was a faculty member of the same university with sufficient experience in the field of qualitative research, with some studies and publications on qualitative research, participated and taught in some workshops in this area. The participants were coded in chronological order. The sampling was continued until there were no new findings. In other words, it was continued to obtain data saturation. Finally, the number of participants reached 16. Whenever there was an ambiguous concept after the code extraction, another interview was arranged to clarify it, so that 20 interviews were conducted with 16 participants. The Colaizzi's phenomenological method was applied for data analysis.

The data were analyzed using the Colaizzi's method as follows: First, after the interviews were conducted, the recorded interviews were listened to several times and written down word by word. The transcribed interviews were then studied several times to obtain the perceptions of the interviewees. Then, the information related to the phenomenon was underlined. Third, a concept from every sentence was extracted to reveal its main idea. During this step, we constantly tried to maintain the relevance of the extracted concept with the original sentence, as well as their relationship. After the codes for each interview were extracted, a following interview was conducted. Fourth, the researcher studied the extracted concepts carefully and categorized them as thematic categories or main concepts, based on their similarity. Fifth, to have a comprehensive description of the studied phenomenon, the researcher placed different thematic categories with similar meanings in larger thematic categories to obtain the main concepts. The final step was validation of the data by referring to each participant and asking about the results (15).

To enhance the rigor in the study, two criteria of dependability of credibility were applied. For credibility, the results and the extracted codes were referred to the participants, and their approval validated the results. Moreover, the same data were referred to an expert of qualitative researches to validate the results. For dependability, the researchers explained the research process and the method of obtaining the results in details to help other researchers in this regard. A limitation of our study was that we included the faculty members based on their own remarks on having experience in recitation of the Quran and meditation on its verses. A strong point in the study was that we performed a quantitative study, and obtained a deep understanding of the phenomenon and the subject.

**Table1.** Characteristics of the Participants

Participant’s Number	Educational Level	Field of Study	Gender	Age
**1**	Ph.D.	Immunology	Male	41
**2**	Ph.D.	Anatomy	Male	39
**3**	Ph.D.	Nursing	Female	50
**4**	Ph.D.	Environmental Health	Male	55
**5**	Specialist	Anesthesiology	Female	42
**6**	Ph.D.	Genetics	Female	45
**7**	MSc	Midwifery	Female	51
**8**	Specialist	Infectious Diseases	Male	39
**9**	Sub-specialist	Gastrointestinal Diseases	Male	48
**10**	Specialist	Internal Diseases	Male	54
**11**	Ph.D.	Microbiology	Male	44
**12**	Ph.D.	Medical Physics	Male	39
**13**	Ph.D.	Environmental Health	Male	34
**14**	Ph.D.	Environmental Health	Female	30
**15**	Ph.D.	Medical Information	Female	33
**16**	Ph.D.	Medical Physiology	Male	39

### 3.1. Ethical Considerations

After the participants were provided with the information regarding the purpose and importance of the study, informed consents were taken and they were free to leave the study whenever they desired. The researcher fully introduced herself to the participants and provided her profile. The permission to record the data was obtained and the secrecy of the data was stressed. During the interviews, participants were provided with privacy and comfort. Demographic information of all participants was kept completely confidential in all phases of the research. The participants were informed of the issues extracted from each interview, and provided with the results of the study on request. Moreover, to avoid any biases during the interviews and data analyses, the researcher disregarded her previous information, thoughts, and subjective knowledge about the issue (bracketing), and avoided reviewing the relevant literature before collecting the data. In addition, the study was approved by the Ethics Committee of the university with a code of 67657/200.

## 4. Results

The data was collected from 20 interviews with 16 participants. Data analysis showed 244 codes, and three main categories were extracted from the codes, including the importance of nutrition in the Quran (referring to certain fruits, vegetables and foods, illustrating heavenly foods and fruits, venerating some fruits and vegetables, nutritional recommendations, the healing power of honey, and the effects of fruits and vegetables mentioned in the Quran on physical and social health); reasons of paying attention to lawful (halal) and unlawful (haram) materials (religious slaughter, wine, meats, consequences of haram edibles consumption, general expression of halal and haram terms); and fasting (fasting and physical health, fasting and mental health).

### 4.1. Importance of Nutrition in the Quran

Participants pointed out the Quran references to certain fruits, vegetables and foods, illustrating heavenly foods and fruits, venerating some fruits and vegetables, nutritional recommendations, healing power of honey and the effects of fruits and vegetables mentioned in the Quran on physical and social health. The faculty members referred to various fruits mentioned in the Quran, and some of them referred to the availability of some fruits at the time, the emphasis placed on some drinks by the Quran, the preference of vegetables and fruits to any other edibles in the Quran, and Allah’s exemplification of some of His blessings. The eighth participant considered illustration of fruits as a sign from Allah and said: “I think mentioning fruits in the Quran is because of two reasons; one is that they are used as foods, and the other is that they are a sign of theology and people are recommended to use the blessings of Allah. Fresh fruits can prevent cancer and are rich in antioxidants. Therefore, I think it is an appropriate recommendation mentioned in the Quran”.

Participants referred to the illustration of fruits and foods in the Quran, and considered foods in this world as a small manifestation of heavenly and divine foods. The 12th participant talked about the difference between heavenly blessings and similar names in this world: “That Allah talks about streams with flowing honey and milk is probably for clarifying things. Heavenly blessings are not comparable with the things we use in this world … Allah wants to give a general picture of heaven and its blessings”.

Another perception by the faculty members was related to the veneration of some fruits and vegetables in the Quran. The 10th participant said: “In addition to His swear on olives, He has mentioned it as a holy tree”. Faculty members referred to the nutritional recommendations mentioned in the Quran including the emphasis on cautiousness about choosing foods, paying attention to foods in various aspects, and avoiding overeating. The 10th participant stated: “The Quran has very nice points. For instance, a verse says: ‘Then let man look to his food. The issues perceived from this verse are great. One is that a person must be careful in choosing the food and whether it is halal and clean”.

Participants believed that fruits and vegetables mentioned in the Quran have positive influences on social and physical health. They particularly referred to the numerous benefits of the foods that Allah has sworn on in the Quran, as well as experiences of using the foods mentioned in the Quran, including honey for therapeutic uses, in their personal lives and in conducted researches. The eighth participant talked about the effects of fresh fruits on preventing cancer: “Fresh fruits are important factors for maintaining the body health, providing it with various vitamins and antioxidants, and preventing cancer”.

The ninth participant referred to the positive impact of fruits on the body: “These fruits have positive effects on the body. Figs are laxative and we recommend them to those with constipation. Olives are also laxative. Grapes have different properties…Grapes can be used in different seasons. Sour grape juice and its leaves can be used as well. They can also be consumed as raisins. Grapes are used in various forms. The alcohol extracted from grapes is illegitimate, but we use medical and industrial alcohol for various applications”. The third participant talked about researches on olive leaves and their effects on herpes: “I myself did a research on the effect of olive leaf extract, showing that it is effective on herpes treatment”.

The second participant explained the destruction of cancerous cells in vitro by antioxidants present in figs, dates and olives: “There are high amounts of antioxidants in dates as well, but there are much more in figs. They will completely destroy cancerous tumors in vitro if we purify their extract from figs. An anticancer drug is to be made using this extract”. Talking about personal experiences related to the healing power of honey, the third participant said: “What I've experienced is that honey is effective on many diseases that may not respond to drugs, besides each drug can have its own side effects”.

### 4.2. The Reason for Some Edibles Being Halal and Haram

Participants mentioned the reason for some edibles being halal and haram, including religious slaughter, wine, and meats (animals with halal meat in the Quran, pig’s meat being haram, consequences of haram foods consumption, and general expression of halal and haram terms).

They referred to permitted and prohibited items mentioned in the Quran, emphasized on the health of meat obtained by religious slaughter and the prevalence of diseases in countries using nonreligious slaughter, and considered chapter of Al-Ma’idah in the Quran as a chapter about halal and haram cases, religious slaughter, and hunting. The seventh participant put an emphasis on the effects of religious slaughter on the meat health as well as human physical health, and referred to the approval of religious slaughter by modern medical sciences and stated: “One thing is bursting forth of the pulsating blood from the animal during slaughtering that is very important for us, Muslims. It is sometimes seen that meat devoid of this blood is more hygienic, and gets less rancid and infected. In many places or tribes, meat is consumed as carrion, possessing this blood in the animal muscles. This involves infectious diseases or other problems that we may be unaware of, including the mad cow disease”.

In addition, participants referred to the prohibition of alcoholic drinks in the Quran, the risk of alcohol presence in the body in medical assessments, and finally its adverse effects on the liver, kidneys, brain, and the overall health of body and soul. The eighth participant elaborated the prohibition of alcoholic drinks: “Consumption of alcoholic drinks is a factor that, we know, can cause numerous damages, including gastric cancer, and particularly hepatic cirrhosis, which is an acute hepatic disease resulting in dementia. A person’s thinking ability decreases due to intoxication by alcohol, and he or she may do actions not compatible with human dignity, or harm his or her body in that condition”.

Participants talked about animals with halal and haram meats in the Quran, referred to four domestic animals, the accordance of their order in the Quran, their importance in Iranian traditional medicine, and healthfulness of fish meat in the Quran. Moreover, they talked about the prohibition of pig meat in the Quran and attributed it to the effect of pig meat on physical and psychological status of humans, its high fat percentage, and existence of parasites in this meat. They also stated that pig is a symbol of filthiness in the West. They stressed that the meat is haram even if the medical problems are eradicated, and that the prevalence of infectious diseases and diseases related to pig meat is lower among Muslims. The 12th participant said: “In the western culture, for giving an example of a physically or morally filthy person, pig is mentioned”.

Concerning the consequences of haram foods consumption and its effects on body, soul, and spirituality, participants noted the side effects of pig meat consumption and alcoholic drinks in the western countries as well as social consequences of alcoholic drinks consumption. About the effect of nutrition and paying attention to the impacts of halal and haram foods on personality and upbringing of children, the second participant said: “Nutrition during pregnancy affects the future of the baby …. It is important whether the parents followed the rules of halal and haram".

Participants pointed out some issues mentioned in the Quran as general expressions of halal and haram terms, including the difference between halal and tayeb (good), recommendation of the Quran to choose tayebs among halals, considering halal a physical and spiritual matter, and halal and haram being in line with human common sense and nature. As the 13th participant noted: “It is always said that there is a reason for everything prohibited or recommend in the Quran. When it is haram, it has some damages for humans”. The 16th participant said: “Anything that causes problems for human health is forbidden in Islam”.

### 4.3. Fasting

Participants elaborated two physical and spiritual aspects of fasting, and emphasized on the usefulness of fasting on the overall body health and the role of relaxation therapy or forbearance in some gastrointestinal diseases. Regarding the effect of fasting on enhancing mental and physical health, the eighth participant said: “Fasting creates such a strong ambition in a person helping him to preserve himself against carnal desires including eating, drinking, and many diseases. With fasting, signs and symptoms decrease particularly in overweight patients, those with nervous colics, and nervous pains”. In addition, some of the participants did not consider the physical aspects of fasting. They noted that psychical health is not mentioned in the Quran when it talks about fasting, and the purpose is to strengthen the soul and increase the piety. Pointing out this matter, the 15th participant stated: “The reason of fasting mentioned in the Quran is not physical; the Quran says that it is for enhancing your piety” (Table 2).

**Table 2.** Primary and Main Categories in the Faculty Members’ Perception of Nutritional Health in the Quran

Main Categories	Arrangement of the Concepts Inside the Thematic Categories	Primary Codes
**Importance of nutrition**		
	Referring to certain fruits, vegetables and foods in the Quran	1-29
	Nutritional recommendations	30-39
	Healing power of honey as well as the effect of fruits and vegetables mentioned in the Quran on physical and social health	41-63
**Reason for some foods being halal and haram**		
	Religious slaughter	64-70
	Wine	71-85
	Animals with halal meat in the Quran	86-91
	Pig’s meat being haram	92-98
	General expression of halal and haram terms	99-107
	Physical consequences of haram edibles consumption	108-118
	Spiritual consequences of haram edibles consumption	119-224
	Social consequences of haram edibles consumption	225-230
**Fasting**		
	Fasting and physical health	231-239
	Fasting and mental health	240-244

## 5. Discussion

The results of this study revealed medical university faculty members’ perception of nutrition mentioned in the Quran. The results showed that faculty members’ perceptions included referring to certain fruits, vegetables and foods, illustrating heavenly foods and fruits, venerating some fruits and vegetables, nutritional recommendations, and effects of fruits and vegetables mentioned in the Quran on physical and social health (first category), and these are consistent with Quranic verses and texts.

According to the results, faculty members perceived that the Quran has referred to some fruits, vegetables, and foods. The results of a study by Shafaghat showed that 18 fruits and plant species from 17 strains and 18 families have been mentioned in the Quran (16). In some verses, the Quran has referred to orchards and fruits without naming any type of fruit; for example, in verses 25-30 in the Abasa chapter (10). The Quran has mentioned some foods in different occasions; for example, olive (At-Tin: 1, Al-An’am: 141, etc.), fig (At-Tin: 1), cucumber, lentil, garlic, onion (Al-Baqarah: 16), grape (An-Nahl: 67, Al-Mu’minun: 19, etc.), bread (Yusuf: 36), pomegranate (Al-An’am: 99 and 141), squash (As-Saffat: 144 and 146), banana (Al-Waqi’ah: 29), vegetables (Abasa: 28), meat (Hud: 69), etc. (17). Some of the participants stated that people at that time were more familiar with these fruits, which is wrong. As Ghorbani mentioned, some people may think that because the ancient people were familiar with these fruits, when the Quran was sent to the Prophet, they were emphasized in these verses. However, we can see that the reason is beyond it, considering the global nature and eternity of the Quran as well as the depth of its interpretations (9).

In addition, the faculty members perceived the illustration of fruits and foods, and this is consistent with Quranic verses. In total, the term “fruit” (fakehah in Arabic and its derivatives) has been used 19 times, 13 of which are related to the use of fruits by the pious and righteous people in heaven (10). The Quran mentions that fruits like grapes, dates, figs, olives, and pomegranates are heavenly fruits and gifts from Allah (8). In addition, these fruits are mentioned more frequently among the heavenly foods because of their nutritional importance, their role in freshness and vitality of humans, and their easy digestion and absorption, confirmed not only scientifically but also experientially (9). According to the Quran, the importance of plants in human nutrition is much more than other edibles, so that Allah has preferred fruits to meats in expressing heavenly foods and blessings (18).

Veneration of some fruits and vegetables was another concept extracted from the faculty members’ perception. Fesharaki et al. stated that the importance and roles of plants can be perceived through the Quranic view on plants (18). As swearing to foods is one of Allah’s swears in the Quran, swear of the Creator to His creation indicates a special respect and admiration by the Creator of the universe for the rules of life including foods and nutrition. He orders people to use healthful and clean foods and blames those who have deprived themselves and others of such foods (4). In addition, in 19 cases the term “fruit” (fakehah and its derivatives) has been used, which can be a clear point about the value of fruits and vegetables from the Quranic point of view. Apart from the verses in which the topic of fruits has been presented generally, there are other verses in the Quran that have mentioned some special fruits including grapes, pomegranates, figs, dates, and olives as a blessing and a favor from Allah (10). According to the verses in the Quran, these fruits are divine gifts and heavenly fruits from Allah (16).

Faculty members referred to the nutritional recommendations in the Quran, and this finding is in accordance with Quranic verses. In the Abasa chapter, verse 24, it has been mentioned that man has to watch the foods he eats carefully and thoughtfully. It means that people have to be very careful about their nutrition (18). Participants of this study interpreted this care about foods from various aspects, including being clean, halal, healthful, and fit. In the Abasa chapter, verses 25-32, eight different types of vegetal foods have been named, suggesting the importance and priority of plants and fruits in human nutrition (9). Plants are at the base of the food pyramid of living organisms and are the main providers of nutrients for humans (19). The remarkable emphasis placed by the verses in the Quran on earth and vegetation, and precedence of fruits to meats is another reason for preference of this nutritional group to meats (9). The foods mentioned in the Quran have many benefits; for instance, olive oil has the best protective role in atherosclerosis. Researchers currently say that this oil contains significant amounts of nine fatty acids, while its benefits have been mentioned in the Holy Quran for years. It is more likely that there are much more unknown benefits in olive oil and other nutrients mentioned in the Quran, which can be discovered in future (17). On the other hand, Allah in the Quran has forbidden humans from extravagance and says in the Al-A’raf chapter, verse 31, that Allah does not like those who are extravagant (20). Eating has to be moderate and sufficient, since overeating and extravagance endanger the human health (8). On the other hand, faculty members did not refer to the milk of livestock in the Quran, while verse 21 in the Al-Mu’minun chapter states: “And most surely there is a lesson for you in the cattle: we make you drink of what is in their bellies, and you have in them many advantages and of them you eat” (21).

Faculty members pointed out the healing power of honey and effects of fruits and vegetables mentioned in the Quran on physical and social health. Fesharaki et al. found that plants, besides being used as foods by humans, have some medical applications as drugs and for treatments (18). The Quran is one of the best reference books describing the roles of plants in treating some diseases in the chapters of Al-Mu’minun, Ar-Rahman, Al-Baqarah, and Al-An’am (17, 22). According to these Quranic lessons, these medicinal plants have been widely used in the Islamic medicine as major sources of drugs for treatment of many diseases. Honey, as a product of various plant species, is described in the Quran for its medicinal benefits (17). Ginger is another herb mentioned in the Quran, and verse 17 in the Ad-Dahr (Al-Insan) chapter says: “and they shall be made to drink therein a cup, the admixture of which shall be ginger” (22). Several studies conducted on olives, dates and grapes indicate that these three sufficiently satisfy human needs for sugar, fat, minerals and vitamins favorably, and for proteins relatively. Moreover, these fruits also have therapeutic effects and are used in treatment of some diseases. Finally, the last point that can clarify the value of fruits in terms of human health is verse 15 in the Muhammad chapter. Allah in this verse has mentioned the opportunity to use these blessings as awards for the pious and heavenly people (10). In another study on biochemical compounds and nutritional roles of the foods mentioned in the Quran, stated that virtually, all the foods known today as perfect foods have been mentioned in the Quran, and their benefits have occasionally been described. As far as it is known about the nutritional role of the foods mentioned in the Quran, these foods play important roles in health and disease prevention (22).

Honey, mentioned in the Quran as a healer and described for its medicinal aspects (22), is a good source of antioxidants, plays a big role in preventing cancer and heart diseases, and circulates in the blood quickly. When mixed with appropriate amount of water, honey circulates rapidly in the blood, aids the brain (with the largest amount of sugar consumption in the body) through releasing sugar, and prevents fatigue. Moreover, it has been confirmed that each type of honey has the same characteristics as the plant sap and can be effective in improving specific diseases (23).

Ajibola et al. stated that foods are consumed for nutrition, metabolic activities, growth and healthy life, and regular consumption of honey has all these benefits. In fact, honey serves as a full meal and its consumption as food and medicine results in nutritional and therapeutic benefits. The use of honey in children infants diets and has been reported in the literature, and health indicators of biomarkers have been observed by various researchers (24). Faculty members emphasized the importance of being halal and haram in the Quran, including religious slaughter, wine, meats (animals with halal meats in the Quran, pig meat as haram), consequences of haram edibles consumption, and general expression of halal and haram terms in the Quran (second category).

According to the Quranic texts, one of the scientific wonders of the Quran is forbidding unsanitary and dangerous foods that are harmful for the human body and mind. This was stated when medical sciences were not advanced and there were no modern tools for diagnosing the diseases pathologies. One of the cases forbidden in the Quran is consumption of carrion (11). As stated in verse 3 of the Al-Ma’idah chapter: “Forbidden to you is that which dies of itself, and blood, and flesh of swine …” (20). The halal brand is not only considered in Islamic countries, but is also popular among non-Muslims as a symbol of healthful food. In medicine, the more blood is removed from meat, the healthier it will be, and this flow of blood out of meat is performed in the best way in Islamic slaughter, compared to other popular methods in the world (25). Moreover, cautiousness about consumption of alcoholic drinks has been recommended in several verses, and in at least three verses people have been strongly warned of alcoholic drinks consumption (1). Kamarulzaman and Saifuddeen in their study stated that prohibition of alcoholic drinks and narcotic drugs decreases risky sexual behaviors associated with drunkenness (5).

Faculty members’ perceptions of meats (animals with halal meats in the Quran, pig meat as haram) were among the other results of the present study. The Quran has referred to animals with halal meats 16 times, fish meat two times, beef two times, milk two times, and honey once (4). Verse 5 in the An-Nahl chapter says: “And He created cattle for you … And of them do you eat” (20). Besides recommending the consumption of meat to satisfy bodily needs, Islam has forbidden consumption of meats of some animals. Eating pig meat has been forbidden in four verses. This repetition signifies the importance of this command and confirms abstinence from pig meat. Despite the efforts made to raise healthy pigs, they still confess that consumption of pig meat can be dangerous for human health. One of the reasons for this ban on pig meat is that it causes moral corruption and lack of prejudice in the consumer (20). The Quran directly invites us to eat fish and Halal meat, mentions them as refreshing divine blessings of life, and asks us to eat them, as saying: “lawful to you is the game of the sea and its food (fish) (Al-Ma’idah: 96) (20).

Consequences of haram were among the findings that faculty members perceived. References show that pigs are unique in terms of sexual behavior and they are so-called non-prejudiced, since they, unlike other animals, do not show any reaction to mating of their mates with other pigs. Therefore, eating pig meat can result in a transfer of this behavior, a decrease in human traits, illegal sexual relationship, and dissemination of prostitution. One of the reasons for considering pig meat as haram is that this animal eats filthy and contaminated foods. Despite the existence of common diseases between humans and pigs and dangerous parasites in pig meat, some persons who have a tendency to consume the meat try to downplay the danger, through suggesting raising the animal hygienically (19).

In general, some basic principles are described in the Quran so that all foods, except for those clearly stated in the Quran, are lawful. Strong emphasis has been placed by Islam on cleanliness, spiritually and nutritionally (8). Nutritional rules in the Quran are classified as halal and haram (1). The Quran has mentioned three characteristics for food hygiene: being uncontaminated, clean (not decayed), and halal (not haram) (Al-Baqarah: 172, Al-Ma’idah: 88) (26).

Faculty members’ perceptions of fasting included fasting and physical health as well as fasting and mental health (third category). The Holy Quran in the Al-Baqarah chapter: 83 reads: “|You who believe! Fasting is prescribed for you, as it was prescribed for those before you, so that you may guard (against evil)” (20). From the medical perspective, fasting includes spiritual and physical components. Gavrankapetanovi discussed the aspects of fasting and its positive characteristics regarding a healthy lifestyle and preventing many diseases (27). It has been currently proved that Islamic fasting increases a person’s resistance to psychological needs, and ultimately leads to physical and mental health. In the past two decades, researchers have found that forbearance to eating and drinking along with changes in sleep patterns and time of eating and drinking can make some changes in the physiology and functions of the body hormonal system. If the changes are desirable, fasting can be used to improve the performance of body systems. Although, patients or those at risk have to be prohibited from fasting. One of the main effects of fasting is physical health. Fasting in Ramadan along with changes in sleep patterns can affect the secretionary patterns of various hormones. Investigation on the effects of this type of worship can be valuable in various aspects and solve physical and psychological health problems (28). Furthermore, the results of Azizi et al. study on fasting and health in Islam showed that fasting has desirable effects on overall health and some diseases (29). It helps in relieving stomach ulcers and metabolic disorders. More recently, our research showed that Islamic fasting can improve the human immunity. Excessive eating leads to obesity, which is associated with more dangerous diseases including cancer, coronary artery disease, hypertension, stroke, liver cirrhosis, and various endocrine and metabolic disorders (28).

Faculty members in medical universities perceived that Allah has made some recommendations on improvement of human nutrition and the therapeutic role of nutrition, considering the undeniable impact of nutrition on physical, mental, and social health. What has been mentioned in the Quran is based on particular logics and reasons, and some of these reasons have been achieved by human sciences, while some others have remained unknown. Although we do not know the reasons for many things in the Quran, we consider it as the foundation. Therefore, following the instructions of the Quran can help the human holistic health.
